# *TP53* IVS3 16 bp Variant and Breast Cancer Risk in Western Mexican Women: A Case–Control Study

**DOI:** 10.3390/cimb47090744

**Published:** 2025-09-11

**Authors:** Mariana Araiza-Guzmán, Bricia M. Gutiérrez-Zepeda, Ana M. Saldaña-Cruz, Ingrid B. Montoya-Delgado, Diana Rubio-Delgado, Pablo Benítez-Villa, Diana M. Hernández-Corona, Adrian Daneri-Navarro, Alicia del Toro-Arreola, Jazmin Márquez-Pedroza, Antonio Quintero-Ramos, Betsabé Contreras-Haro

**Affiliations:** 1 Licenciatura en Médico Cirujano y Partero, Centro Universitario de Ciencias de la Salud, Universidad de Guadalajara, Guadalajara 44340, Mexico; mariana.araiza4344@alumnos.udg.mx; 2 Doctorado en Genética Humana, Departamento de Biología Molecular y Genómica, Centro Universitario de Ciencias de la Salud, Universidad de Guadalajara, Guadalajara 44340, Mexico; bricia.gutierrez2314@alumnos.udg.mx (B.M.G.-Z.); ingrid.montoya9883@alumnos.udg.mx (I.B.M.-D.); 3 Laboratorio de Inmunología, Departamento de Fisiología, Centro Universitario de Ciencias de la Salud, Universidad de Guadalajara, Guadalajara 44340, Mexico; adrian.daneri@academicos.udg.mx (A.D.-N.); alicia.deltoro@academicos.udg.mx (A.d.T.-A.); 4 Instituto de Terapéutica Experimental y Clínica, Departamento de Fisiología, Centro Universitario de Ciencias de la Salud, Universidad de Guadalajara, Guadalajara 44340, Mexico; miriam.saldac@academicos.udg.mx; 5 Departamento de Oncología Médica, UMAE, HE, Centro Médico Nacional de Occidente, IMSS, Guadalajara 44340, Mexico; arubio@oncologia.org.mx (D.R.-D.); pbenitez@oncologia.org.mx (P.B.-V.); 6 Departamento de Ciencias Biomédicas, Centro Universitario de Tonalá, Universidad de Guadalajara, Guadalajara 45400, Mexico; diana.hcorona@academicos.udg.mx; 7 División de Neurociencias, Centro de Investigaciones Biomédicas de Occidente, Instituto Mexicano del Seguro Social, Guadalajara 44340, Mexico; jaz180688@gmail.com; 8 Unidad de Investigación Biomédica 02, UMAE, Hospital de Especialidades, Centro Médico Nacional de Occidente, IMSS, Guadalajara 44340, Mexico

**Keywords:** rs17878362, p53, *TP53*, breast cancer

## Abstract

Background: Mutations in the *TP53* gene can alter its tumor suppressor functions, thereby promoting oncogenic activity. The *TP53* IVS3 16 bp genetic variant overlaps with nucleotide sequences that can alter regulatory structures, potentially affecting its function. The aim of the present study was to evaluate the association between *TP53* IVS3 16 bp genetic variant and the risk of breast cancer (BC) in women from western Mexico. Methods: The study included 220 women diagnosed with BC and 198 cancer-free controls. Clinical and demographic data were collected through structured questionnaires and verified with medical records. Genotyping of the *TP53* IVS3 16 bp genetic variant was performed using polymerase chain reaction (PCR) and visualized on 6% polyacrylamide gels. Results: Compared to controls, women with BC more frequently reported a family history of the disease and menopausal status (*p* < 0.05). Genotypic analysis revealed that carriers of the D/I genotype and the combined D/I + I/I genotypes were associated with a reduced risk of BC in codominant (OR = 0.53; 95% CI 0.32–0.88) and dominant (OR = 0.57; 95% CI 0.35–0.93) models. Conclusions: The D/I and D/I + I/I genotypes in codominant and dominant models showed a lower risk against BC. More studies are needed to confirm these findings.

## 1. Introduction

According to GLOBOCAN, breast cancer (BC), with 2.3 million new cases, ranks as the fifth leading cause of cancer-related mortality [1]. The primary risk factor for developing BC is being female, alongside other nonmodifiable factors such as genetic variants [2].

Tumor protein 53 gene (*TP53*) is a widely studied gene with tumor suppressor actions; it is located on chromosome 17 (17p13.1) with 11 exons and 393 amino acid residues, which encodes a 53 kDa nuclear phosphoprotein of 53 kDa (p53) [3]. Wild type p53, best known as the “guardian of the genome”, is involved in multiple gene expressions that regulate metabolism, cell cycle arrest, senescence, DNA repair, and apoptosis [3]. Ten isoforms of p53 have been identified, many of which differ in the N-terminal region that contains the HDM2 (Human Double Minute 2) binding site. This region targets p53 for proteasomal degradation via E3 ubiquitin ligase and is essential for regulating p53 stability [4]. Transcription of p53 messenger RNA generates two proteins with a distinct amino terminus: the canonical full-length p53 and the ∆40p53 isoforms; the last differ at the N-terminus, with ∆40p53 lacking the first 39 amino acids that include the HDM2 binding domain [5]. The ∆40p53 isoform is the most highly expressed in BC and, when overexpressed, may act as a dominant negative inhibitor of p53, reducing its growth-suppressive and pro-apoptotic functions. The balance between full-length p53 and ∆40p53 is therefore critical for normal cell proliferation [5].

**Figure 1:**
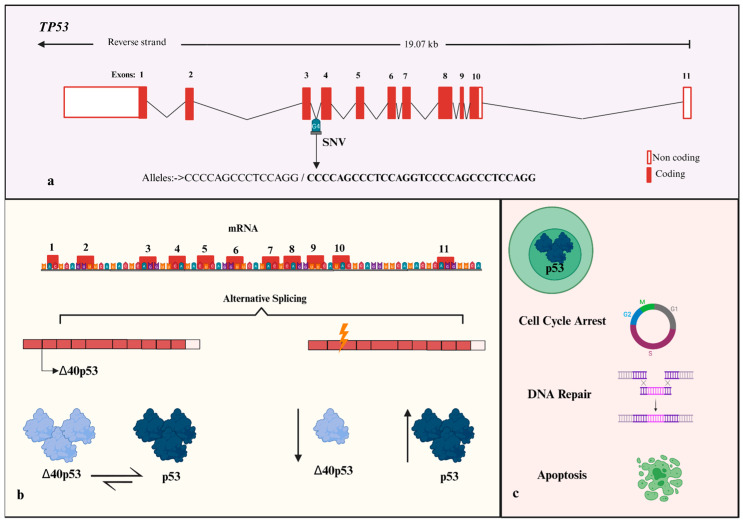
Effect of 16 bp duplication in intron 3 of *TP53*. (**a**) Intron 3 harbors G-quadruplex (G4) structures that regulate intron 2 splicing [18]. The 16 bp duplication overlaps with this region, modulating the balance between the p53 and Δ40p53 isoforms. (**b**) DNA damage due to the presence of 16 bp duplication, can altered splicing and change the Δ40p53:p53 ratio [6]. (**c**) An imbalance between these isoforms enhances p53 activity in cell cycle arrest, DNA repair, and apoptosis. In BC, duplication has been associated with a lower Δ40p53 proportion, leading to higher levels of p53 and a more favorable clinical outcome. This hypothesis is based on previously described mechanisms and requires further functional validation. Figure created in BioRender. Bravo, K. (2025). https://BioRender.com/2ouf39i (accessed on 11 August 2025).

Mutations in *TP53* that influence splicing and interfere with the binding of splicing factors, often lead to the generation of abnormal mRNA transcripts, or the isoforms expression altered [6], reducing tumor suppressive functions [7]. Almost 100 different TP53 mutations have been identified, including missense mutations, truncated mutations, and in-frame and splice mutations [5]. In BC, *TP53* is the most frequently mutated gene [8], and it has been estimated that approximately 90% are somatic mutations and contribute to sporadic tumors, while the remaining 10% are germline mutations [9,10]. Likewise, there is a high frequency of de novo pathogenic variants in *TP53* associated with Li–Fraumeni syndrome (LFS), similar to BC [11]. The genetic variant 16bp insertion (I) (ACCTGGAGGGCTGGGG, *TP53* IVS3 16 bp genetic variant, rs17878362) in intron 3 [12] overlaps their nucleotides and decreases nucleotides in the intron 2 [13]. Exon 3 and intron 3 are relatively short—21 bp and 112 bp, respectively—and even small sequence variations (e.g., a 15% increase in intron length) may significantly affect splicing efficiency [6]. The 16 bp duplication (insertion allele) causes an alteration in the conversion process of precursor messenger RNA into mRNA (see Figure 1). Previous studies in different populations and other ethnic groups, but none in the Mexican population, have analyzed the association between the *TP53* IVS3 16 bp variant and the risk of BC, with controversial results [12,13,14,15,16]. Some of the studies were performed in European and Asian populations [17], but there is a lack of information in the Latino American population. According to the findings, we hypothesize that the *TP53* IVS3 16bp variant may affect the risk of BC in our population. Given the central role of *TP53* in maintaining genomic stability, elucidating the impact of its intronic variants on BC susceptibility, findings from this study may improve personalized risk assessment and guide future molecular diagnostic strategies in oncology. Therefore, the study aimed to evaluate the association between the *TP53* IVS3 16 bp deletion/insertion (D/I) genetic variant and the risk of BC in women from western Mexico.

## 2. Materials and Methods

### 2.1. Subjects and Clinical Setting

We conducted a case–control study involving women over 18 years of age, conformed by 216 women with BC (case group) and 198 healthy women as the control group. The sample size was calculated by a priori power analysis, using the proportion comparison formula and considering an exposure proportion in controls (P_2_) of 0.10 and according to the presence of the insertion variant. A significance level of α = 0.05 and a power of 80% were established. Based on the expected effect of 2 (odds ratio), a sample size of 142 individuals per group was obtained, so the sample size used has a statistical power greater than 80%. The case group included patients with BC diagnosis histologically confirmed by a medical pathologist with invasive breast ductal or lobular carcinoma, and patients who met the criteria of Mexican mestizos, defined according to the Mexican National Institute of Anthropology and History (INAH) [19] as “Individuals born in Mexico, of the third generation including their own and descendants of the original autochthonous inhabitants of the region and individuals who were mainly Spaniards”, were included [20]. The patients were attended to at the BC Medical Oncology Clinic in a tertiary care hospital, the Hospital de Especialidades, Centro Medico Nacional de Occidente, Instituto Mexicano del Seguro Social (IMSS) from January 2022 to August 2023. Furthermore, the case group was paired by age (±5 years) to Mexican mestizo subjects from the same geographical region as the control group. The subjects were recruited via invitation during a clinical visit to the Institute of Experimental Therapeutics of the Outpatient Research Clinic of the University of Guadalajara in the same period, as part of another research, where the investigators explained the goals of the study. For the latter group, they reported no clinical evidence of BC disease in the last year confirmed by mammography. Consent forms were explained to the participants of both groups, case and control, and they were signed by each one.

### 2.2. Clinical Evaluations

Two trained researchers took a blood sample and performed a physical examination. In addition, all subjects were interviewed in a structured way, where clinical characteristics such as BC family history and lifestyle characteristics were recorded.

Alcoholism was defined as more than 80 g of alcohol per day, and the patients were asked about the quantity of alcohol units they consume and their frequency of drinking [21]. Current tobacco use was defined as an adult who currently smokes or smoked 100 cigarettes in his life. Patients were asked about the number of cigarettes they smoke and their frequency of tobacco use [22]. Menopause was defined as at least 12 months of amenorrhea caused by a decrease in female sex hormones secondary to natural causes, excluding diseases or surgical menopause.

Furthermore, the clinical characteristics of the tumor were obtained from a pathology report. The staging of BC was performed according to the American Joint Committee on Cancer (AJCC 9th) [23] based on tumor size, lymph node evaluation, and evidence of metastatic disease [24]. The tumor phenotype was classified according to immunohistochemical expression of hormone receptors (ER^+^/PR^+^), overexpression of the human epidermal growth factor receptor 2 (HER2), and Ki-67 proliferation index [25]. Hormone receptor expression was considered positive if the stains were 1% or more of the nuclei [25], and the proliferation index Ki67 was measured by the relationship between positive tumor cells and total cells, reported as a percent [26]. HER2 was considered positive if tumor cells showed intense circumferential membranous staining in more than 10% and were reported as HER2^+++^. If the stain was weak-to-moderate in the complete membrane that reported HER2^++^, an in situ hybridization (ISH) was carried out. An incomplete stain of less than 10% was reported, such as HER2 + or HER2 0+, which was considered negative [26].

Histological grade was evaluated using the Scarff-Bloom-Richardson classification system (NCCN, 2024) [25].

A sedentary lifestyle was considered as women who did not exercise regularly at least 30 min/day for 4 days as a minimum. Body mass index (BMI) was calculated using the Quetelet formula (weight (kg)/height (m)^2^). The patients were classified as normal weight (18.5–24.9 kg/m^2^), overweight (25–29.9 kg/m^2^), and obese (≥30 kg/m^2^) [27].

### 2.3. Genomic DNA Isolation and Genotyping

Whole blood samples were collected in tubes containing sterile ethylenediaminetetraacetic acid (EDTA) with anticoagulant, BC and RG. Genomic DNA (gDNA) was obtained from total peripheral blood using the modified Miller method, previously described [28]. Identification of the *TP53* IVS3 16 bp genetic variant was carried out by PCR using modified primer sequences 5-CTG AAA ACA ACG TTC TGG TA-3 (forward primer) and 5-AAG GGG GAC TGT AGA TGG-3 (reverse primer) [14]. PCRs were carried out using 10 ng of genomic DNA in a total volume of 10 μL, containing 1X PCR buffer, 1.5 mM MgCl_2_, 100 mM of each dNTP, 0.3 mM of each primer, and 0.025 U of recombinant Taq DNA polymerase, all Invitrogen reagents (Life Technologies Corporation, Carlsbad, CA, USA). Subsequently, the reaction was carried out on an Aeris thermal cycler (Esco^®^ Lifesciences group, Changi, Singapore) with the following conditions: initial denaturation step at 94 °C for 4 min; followed by 30 amplification cycles of 25 s each, at 94 °C, 54 °C, and 72 °C; and then a final extension at 72 °C for 7 min. Fragments of 118 pb (base pairs, deletion, D) or 134 pb (insertion, I) were obtained. These fragments were visualized at 6% of 29:1 polyacrylamide gel electrophoresis (Golden Bell reagents, Jalisco, Mexico) on an OWL P9DS camera (Thermo Fisher Scientific, Waltham, MA, USA) and stained with silver nitrate (Golden Bell reagents, Jalisco, Mexico). As a quality control, 10% of all samples were repeated and full matching was obtained. The genotypes of the genetic variant were classified into one of the following three categories: wild homozygous (D/D), heterozygous (D/I), and mutated homozygous (I/I).

### 2.4. Ethics

This study protocol was approved by the Research Ethics Committee of the Instituto Mexicano del Seguro Social, Jalisco, Mexico. Approval code: R-2023-1301-150; Approval date: 7 August 2023. The procedures were carried out during the study following the guidelines of the Declaration of Helsinki. All participants willingly agreed to participate and gave their informed written consent to the start of the study.

### 2.5. Statistical Analysis

The Hardy–Weinberg equilibrium (HWE) in the control group was calculated based on the observed versus expected proportions using the chi-square test. Comparison of clinical characteristics between groups was computed by the Student’s *t*-test for means, and chi-square test or Fisher’s exact test for proportions. Crude odds ratios (OR) and their 95% confidence intervals (95% CI) were calculated to analyze the association between the genetic variant and BC. A *p* < 0.05 level was considered significant. Data were analyzed with SPSS v.25 statistical software program (SPSS Inc., Chicago, IL, USA), and the OR and its 95% CI were obtained by EPIINFO ver. 7.2 software (Epi Info™; Atlanta, GA, USA).

## 3. Results

### 3.1. Description of Clinical Variables

We included 220 women with BC as the case group and 198 healthy women as the control group. The determination of complete clinical characteristics was only possible in 216 women with BC, of the 220 included in the present study. BC women had a higher frequency of BC with family inheritance history (FIH) as well as menopause and years of menopause, compared to the control group, where *p* < 0.05. No differences were observed in other clinical characteristics such as age, alcoholism, tobacco use, sedentary lifestyle, and body mass index; both groups behaved very similarly (see Table 1).

Table 2 shows tumor characteristics and treatment related to BC women. The mean duration of the disease was 3.6 ± 5.3 years, and 33.8% of women with BC had clinical stage III or IV. In the same way, most women with BC had a tumor with a histological grade moderately differentiated (73.1%). Related to the objective of treatment (see Table 3), 26.4% received neoadjuvant treatment, and only 1.9% received adjuvant treatment. Anthracyclines, cyclophosphamide, and taxanes were the widely received cytotoxic treatment in BC patients. Most of the patients (40.3%) received aromatase inhibitors as hormone therapy in the same way the hormone therapy was the treatment provided during sampling in the 63.4% of the patients (see Table 4).

### 3.2. Genetic Association

**Figure 2:**
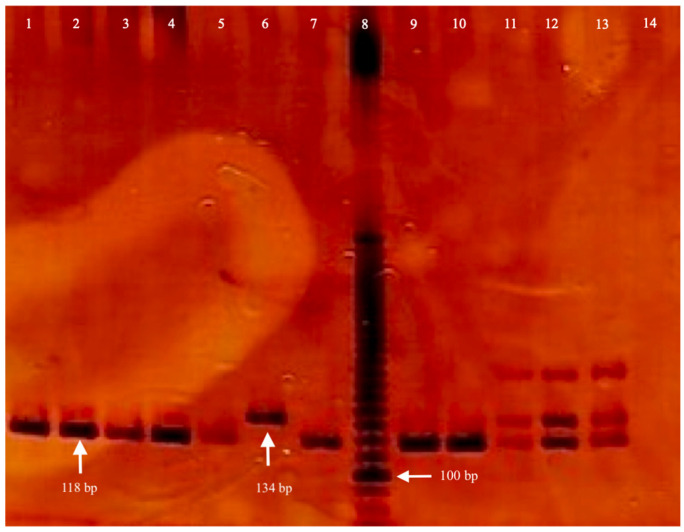
Electrophoresis results of the *TP53* IVS3 16 bp genetic variant. Lane 8 contains a 10-base-pair (bp) molecular weight marker (Invitrogen reagent, Life Technologies Corporation, Carlsbad, CA, USA), with the 100 bp band indicated. Lanes 1–5, 7, 9 and 10 display a single band at 188 bp, corresponding to the wild-type homozygous genotype Deletion/Deletion. Lane 6 shows a single band at 134 bp, representing the homozygous mutated-type genotype Insertion/Insertion. Lanes 11–13 exhibit two bands at 118 bp and 134 bp, indicative of the heterozygous genotype Deletion/Insertion. Lane 14 corresponds to the negative PCR control.

The allele and genotype frequencies for the *TP53* IVS3 16 bp genetic variant were consistent with the HWE in both study groups (*p* > 0.05). The frequency of the D allele was higher in the case group (BC, Table 5), suggesting that this variant may represent the ancestral allele in this population. However, the difference between groups was not statistically significant. Electrophoretic patterns allowed us to identify the three possible genotypes: homozygous deletion (D/D), heterozygous (D/I), and homozygous insertion (I/I) (see Figure 2).

Table 5 shows the D allele and the D/D genotype were the most frequent in both case and control groups. When comparing groups, the I allele showed a trend towards a reduced risk of BC (OR = 0.64, 95% CI 0.41–1.02, *p* = 0.07). This effect was further supported in the heterozygous D/I genotype (OR = 0.53, 95% CI 0.32–0.88, *p* = 0.02) and in the dominant inheritance model (D/D vs. D/I + I/I; OR = 0.57, 95% CI 0.35–0.93, *p* = 0.03).

We stratified the case group (BC) and compared the stratification of patients with BC according to recessive or dominant models and clinical variables. In the dominant model, we found a longer breastfeeding time (months) in patients with the I/I genotype and no differences between other clinical or treatment variables. In the recessive model, carriers of I/I + D/I had a higher frequency of alcoholism. No other differences were found in clinical or treatment variables. Furthermore, the case group was analyzed under dominant and recessive inheritance models to compare with the different clinical characteristics; however, no significant associations were observed with any variable.

## 4. Discussion

The study provides critical information on the association between the *TP53* IVS3 16 bp genetic variant and the risk of BC, as well as an analysis of demographic, clinical, and lifestyle factors in Mexican mestizo women. No association was found between the *TP53* IVS3 16 bp genetic variant and BC. However, carriers of the Ins allele in the co-dominant and dominant model had a lower risk of BC. The results highlight the interaction between genetic predisposition and modifiable risk factors, providing a nuanced understanding of BC in this population.

Our results did not show an association between the genetic variant and BC risk, similarly to the findings reported by Akkiprik [29]. In contrast, Fhagani et al. and Costa et al. report significant associations in the Iranian and Portuguese population [13,14]. Interestingly, Floris et al. found that the Ins allele was associated with a lower risk of BC in patients aged >50 years, which is consistent with our results, although we did not perform an age-stratified analysis [17].

These differences among studies may be explained by variations in genetic backgrounds across populations. In our population, the higher frequency of the D allele could be related to the genetic admixture characteristic of the Mexican population, which is primarily derived from Amerindian, European, and, to a lesser extent, African ancestries [20]. Results from European populations, such as Portuguese and Sardinian cohorts have been variable [14,17], and studies in Asian populations, including Chinese Han women [16], have shown no significant association. These observations support the idea that genetic structure and admixture can influence the impact of this variant on BC susceptibility.

Similarly, when analyzing clinicopathological variables, no association was found in the dominant or recessive model, in agreement with Morten et al.’s research on Australian patients [13] and Hao et al.’s work on Chinese patients [16]. This is contrary to Faghani et al. [15] who found an association with positive nodal status but did not mention the genotype or the model associated with it.

Regarding the analysis of lifestyle and demographic factors in our study between models, carriers of Ins/Ins + Del/Ins in the recessive model had a higher frequency of alcoholism, in line with studies by Floris et al. [17], where the authors reported a strong association between alcohol intake during adolescence and the risk of BC. Ethanol metabolism leads to the production of metabolites that may cause cell death or disruption of cell function disturbances and acts as an enzymatic substrate that is incorporated into oxidative metabolic pathways, increasing reactive oxygen species and consequently causing cellular damage. It also may cause carcinogenic effects through epigenetic effects and retinoid metabolism [30]. In our study, a longer duration of breastfeeding was observed. This finding could suggest a possible interaction between genetic predisposition and reproductive factors due to hormonal changes, reduction in serum insulin levels, and immunomodulation in breastfeeding women [31].

The differences could be attributed to possible differences in their ancestry. Furthermore, in terms of body composition, some studies adjusted or considered in their analysis the body mass index. This may be because adipose tissue produces chronic inflammation and altered adipokine balance, which may lead to further carcinogenesis [32].

The association with lower risk observed in carriers of the insertion allele D/I and D/I + I/I is consistent with a TP53 isoform-balance model. This can be explained by the fact that Δ40p53 modulates the transcriptional activity of p53, and that intronic structural variation can alter the balance between full-length p53 and Δ40p53, thereby reducing the Δ40p53:p53 ratio [33].

A plausible explanation is that the 16 bp duplication shifts the position of the G-quadruplex within intron 3 relative to the intron 2 splice-acceptor site, subtly biasing splice-site choice and transcript stability in favor of full-length [34]. In line with this interpretation, tumor studies such as that reported by Morten et al. [33] in an Australian study on BC tissue found that tumors harboring the intronic 16 bp insertion exhibited higher full-length p53, a lower Δ40p53 mRNA ratio, and better disease-free survival. Greater availability of full-length p53 is expected to reinforce tumor-suppression pathways, promoting more effective cell-cycle arrest and DNA repair and appropriately engaging apoptotic pathways (see Figure 1). Notably, they found no association with hormone-receptor status or tumor size, which is consistent with our analyses [33].

This hypothesis is consistent with previous findings indicating that changes in p53 isoform ratios can significantly influence cell fate decisions in breast tissue. Future functional studies are needed to explore the mechanistic basis of *TP53* IVS3 16 bp variant, its effect on isoform expression, and its potential role as a biomarker for BC susceptibility or prognosis [35].

The *TP53* gene is well-known as the most frequently mutated gene in breast cancer (BC). These results may help to assess cancer risk in BC patients with *TP53* variants and assist clinicians in understanding cancer risk with a more discrete, individualized frame of reference. Our findings require validation in other independent Mexican cohorts and in the context of other known determinants of cancer risk.

The present study has some strengths. Its adherence to HWE supports the genetic validity of the results, while the use of multiple statistical models (allelic, co-dominant, and dominant) provides a comprehensive understanding of genotype effects. To the best of our knowledge, this is the first study that analyzes the 16 bp genetic variant association between the *TP53* IVS3 16 bp variant and BC risk in a female population of western Mexico. It is important to note that this is a single-center study with a relatively small sample size. More studies with larger sample sizes and multi-center studies are needed to confirm these findings and investigate the underlying mechanisms by which this polymorphism may influence the risk of BC.

Although *TP53* variants have been extensively studied worldwide, regarding data on their contribution to breast cancer risk in Latin American populations, and particularly in Mexican women, there are no studies. Given the unique genetic admixture of the Mexican population, extrapolating results from other ethnic groups may overlook population-specific risk associations [20].

Some limitations of the study were as follows: (a) the concentration of the TP53 protein and mRNA levels were not quantified, so it was not possible to corroborate which patients produce protein or a functional mRNA according to their genotype; (b) although the sample size exceeds the statistical power of 80%, it is still a relatively low sample; (c) difficulty in validating the effect of *TP53* IVS3 16 bp genetic variant, due to the lack of functional tests; (d) the results reported in this study are specific to western Mexican women, so it is not recommended that they be extrapolated to other populations, since this is a single-center study design; (e) the obstetric information was not available for the control group; (f) the inclusion of all BC molecular phenotypes and patients were under active cancer treatment; (g) lifestyle differences and environmental confounders; (h) selection bias in case–control recruitment.

## 5. Conclusions

In conclusion, we found that the D/I and D/I + I/I genotypes in codominant and dominant models were associated with lower risk of BC. These findings highlight the importance of understanding genetic contributions within specific populations, as ancestry and environmental factors can modulate cancer risk profiles. Future multi-center studies with larger samples and functional analyses of P53 isoform expression are needed to confirm these results.

Such research may contribute to improved BC risk, evaluating the underlying biological mechanisms, stratification, and personalized therapeutic strategies.

## Figures and Tables

**Table 1:** Comparison of clinical characteristics between patients with BC (case group) versus the control group.

Variable	Case Group *n* ^a^ = 216	Control Group *n* = 198	*p* ^b^
Age (years), mean	55.6 ± 10.1	56.4 ± 12.8	0.5
BC with FIH ^c^, *n* (%)	46 (21.3)	12 (6.1)	<0.001
Alcoholism, *n* (%)	72 (33.3)	60 (30.6)	0.1
Tobacco smoke, *n* (%)	63 (29.4)	0	0.6
Sedentary lifestyle, *n* (%)	108 (54.8)	114 (52.8)	0.7
Pregnancy, *n* (%)	194 (89.8)	-	
Lactation (months), mean	6.6 ± 12.1	-	
Menopause, *n* (%)	187 (86.6)	124 (62.4)	<0.001
Menopause years	40 ± 16.7	35 ± 21	0.03
Body mass index	27.6 ± 4.4	28.5 ± 5.06	0.08
Underweight, *n* (%)	1 (0.5)	3 (1.6)	0.2
Normal, (%)	69 (32.5)	53 (26.7)	0.9
Overweight, *n* (%)	81 (37.5)	79 (41.1)	0.9
Obese, *n* (%)	65 (30.7)	63 (32.8)	0.4

^a^: sample number; ^b^: significance defined by the X^2^ test; ^c^: FIH: family inheritance history.

**Table 2:** Tumor characteristics in patients with BC.

Variable	*n* ^a^ = 216
Disease duration (years), mean	3.6 ± 5.3
Clinical stage, *n* (%)	
I	44 (20.4)
II	99 (45.8)
III	61 (28.2)
IV	12 (5.6)
Histology, *n* (%)	
Ductal	183 (84.7)
Lobulillar	25 (11.6)
Other	8 (3.7)
Histological grade, *n* (%)	
Well-differentiated	24 (11.1)
Moderately differentiated	158 (73.1)
Poorly differentiated	34 (15.8)

^a^: sample number.

**Table 3:** Initial treatment strategies for patients with BC.

Variable	*n* ^a^ = 216
Objective of treatment	
Neoadjuvant, *n* (%)	57 (26.4)
Adjuvant, *n* (%)	4 (1.9)
Palliative, *n* (%)	7 (3.2)
Chemotherapy	
Anthracyclines, *n* (%)	99 (45.8)
Taxanes, *n* (%)	83 (38.4)
Carboplatin, *n* (%)	3 (1.4)
Cyclophosphamide, *n* (%)	85 (39.4)
Hormone therapy	
Tamoxifen, *n* (%)	38 (17.6)
Aromatase inhibitor, *n* (%)	87 (40.3)
Tamoxifen and aromatase inhibitor, *n* (%)	24 (11.1)
Radiotherapy, *n* (%)	14 (6.5)

^a^: sample number.

**Table 4:** Treatment status during sample collection.

Variable	*n* ^a^ = 216
Treatment provided during sampling	
Without treatment, *n* (%)	19 (8.9)
Chemotherapy, *n* (%)	30 (13.9)
Targeted therapy, *n* (%)	31 (14.4)
Hormone therapy, *n* (%)	137 (63.4)
Radiotherapy, *n* (%)	6 (2.8)

^a^: sample number.

**Table 5:** Allelic and genotypic frequencies and their association between the *TP53* IVS3 16 bp genetic variant.

Inheritance Models	Control Group *n* ^a^ (%)	Case Group *n* (%)	OR ^b^ (95%CI ^c^)	*p* ^d^
Allele				
D ^f^	405 (0.92)	349 (0.88)	Reference	
I ^g^	35 (0.08)	47 (0.12)	0.64 (0.41–1.02)	0.07
Co-dominant				
D/D	187(0.85)	151 (0.76)	Reference	
D/I	31 (0.14)	47 (0.24)	0.53 (0.32–0.88)	0.02
I/I	2 (0.01)	0 (0.0)	NS ^e^	0.58
Dominant				
D/D	187(0.85)	151 (0.76)	Reference	
D/I + I/I	33 (0.15)	47 (0.24)	0.57 (0.35–0.93)	0.03
Recessive				
D/D + D/I	218 (0.99)	198 (1.00)	Reference	
I/I	2 (0.01)	0 (0.0)	NS ^e^	0.53

^a^: sample number; ^b^: odds ratio; ^c^: confidence interval; ^d^: significance defined by the X^2^ test; ^e^: no significance; ^f^: Deletion; ^g^: Insertion.

## Data Availability

The datasets presented in this article are not readily available because the data are part of an ongoing study. Requests to access the datasets should be directed to the corresponding authors.

## References

[1] Bray F., Laversanne M., Sung H., Ferlay J., Siegel R.L., Soerjomataram I., Jemal A. (2024). Global cancer statistics 2022: GLOBOCAN estimates of incidence and mortality worldwide for 36 cancers in 185 countries. CA Cancer J. Clin..

[2] Łukasiewicz S., Czeczelewski M., Forma A., Baj J., Sitarz R., Stanisławek A. (2021). Breast Cancer—Epidemiology, Risk Factors, Classification, Prognostic Markers, and Current Treatment Strategies—An Updated Review. Cancers.

[3] Feroz W., Sheikh A.M.A. (2020). Exploring the multiple roles of guardian of the genome: P53. Egypt. J. Med. Hum. Genet..

[4] Bang S., Kaur S., Kurokawa M. (2019). Regulation of the p53 Family Proteins by the Ubiquitin Proteasomal Pathway. Int. J. Mol. Sci..

[5] Guo Y., Wu H., Wiesmüller L., Chen M. (2024). Canonical and non-canonical functions of p53 isoforms: Potentiating the com-plexity of tumor development and therapy resistance. Cell Death Dis..

[6] Marcel V., Tran P.L., Sagne C., Martel-Planche G., Vaslin L., Teulade-Fichou M.-P., Hall J., Mergny J.-L., Hainaut P., Van Dyck E. (2011). G-quadruplex structures in TP53 intron 3: Role in alternative splicing and in production of p53 mRNA isoforms. Carcinogenesis.

[7] Halim F., Azhar Y., Suwarman S., Hernowo B. (2022). p53 Mutation as Plausible Predictor for Endocrine Resistance Therapy in Luminal Breast Cancer. F1000Research.

[8] Marvalim C., Datta A., Lee S.C. (2023). Role of p53 in breast cancer progression: An insight into p53 targeted therapy. Theranostics.

[9] Gallardo-Alvarado L.N., Tussié-Luna M.I., Díaz-Chávez J., Segura Y.X., Bargallo-Rocha E., Villarreal C., Herrera-Montalvo L.A., Herrera-Medina E.M., Leon D.F.C.-D. (2019). Prevalence of germline mutations in the TP53 gene in patients with early-onset breast cancer in the Mexican population. BMC Cancer.

[10] Cárdenas-Sánchez J., Erazo-Valle-Solís A.A., Arce-Salinas C., Bargalló-Rocha E., Piña V.B., Cervantes-Sánchez M.G., Flores-Balcázar C.H., Lluch-Hernández A., Maffuz-Aziz A., Pérez-Sánchez V.M. (2022). Consenso Mexicano sobre diagnóstico y tratamiento del cáncer mamario. Octava revisión. Colima 2019. GAMO.

[11] Schneider K., Zelley K., Nichols K.E., Levine A.S., Garber J., Adam M.P., Feldman J., Mirzaa G.M., Pagon R.A., Wallace S.E., Amemiya A. (1993). Li-Fraumeni Syndrome. GeneReviews®.

[12] Sagne C., Marcel V., Amadou A., Hainaut P., Olivier M., Hall J. (2013). A meta-analysis of cancer risk associated with the TP53 intron 3 duplication polymorphism (rs17878362): Geographic and tumor-specific effects. Cell Death Dis..

[13] Morten B.C., Chiu S., Oldmeadow C., Lubinski J., Scott R.J., Avery-Kiejda K.A. (2019). The intron 3 16 bp duplication poly-morphism of p53 (rs17878362) is not associated with increased risk of developing triple-negative breast cancer. Breast Cancer Res. Treat..

[14] Costa S., Pinto D., Pereira D., Rodrigues H., Cameselle-Teijeiro J., Medeiros R., Schmitt F. (2008). Importance of TP53 codon 72 and intron 3 duplication 16bppolymorphisms in prediction of susceptibility on breast cancer. BMC Cancer.

[15] Faghani M., Ghasemi F., Nikhbakht M., Salehi M. (2011). TP53 PIN3 polymorphism associated with breast cancer risk in Iranian women. Indian J. Cancer.

[16] Hao W., Xu X., Shi H., Zhang C., Chen X. (2018). No association of TP53 codon 72 and intron 3 16-bp duplication polymorphisms with breast cancer risk in Chinese Han women: New evidence from a population-based case–control investigation. Eur. J. Med. Res..

[17] Floris M., Pira G., Castiglia P., Idda M.L., Steri M., De Miglio M.R., Piana A., Cossu A., Azara A., Arru C. (2022). Impact on breast cancer susceptibility and clinicopathological traits of common genetic polymorphisms in TP53, MDM2 and ATM genes in Sardinian women. Oncol. Lett..

[18] Dyer S.C., Austine-Orimoloye O., Azov A.G., Barba M., Barnes I., Barrera-Enriquez V.P., Becker A., Bennett R., Beracochea M., Berry A. (2025). Ensembl 2025. Nucleic Acids Res..

[19] de Pública A.D.I. INAH—The Mexican National Institute of Anthropology and History, Mexico City. https://mexicocity.cdmx.gob.mx/tag/inah/.

[20] González-Quezada B., Creary L., Munguia-Saldaña A., Flores-Aguilar H., Fernández-Viña M., Gorodezky C. (2019). Exploring the ancestry and admixture of Mexican Oaxaca Mestizos from Southeast Mexico using next-generation sequencing of 11 HLA loci. Hum. Immunol..

[21] Soler-Vila H., Galán I., Valencia-Martín J.L., León-Muñoz L.M., Guallar-Castillón P., Rodríguez-Artalejo F. (2014). Binge drinking in Spain, 2008–2010. Alcohol. Clin. Exp. Res..

[22] Tobacco Use—Health, United States. https://www.cdc.gov/nchs/hus/topics/tobacco-use.htm.

[23] American Joint Committee on Cancer, ACS. https://www.facs.org/quality-programs/cancer-programs/american-joint-committee-on-cancer/.

[24] Teichgraeber D.C., Guirguis M.S., Whitman G.J. (2021). Breast Cancer Staging: Updates in the AJCC Cancer Staging Manual, 8th Edition, and Current Challenges for Radiologists, From the AJR Special Series on Cancer Staging. Am. J. Roentgenol..

[25] Guidelines Detail, NCCN. https://www.nccn.org/guidelines/guidelines-detail?id=1419.

[26] Sullu Y., Tomak L., Demirag G., Kuru B., Ozen N., Karagoz F. (2023). Evaluation of the relationship between Ki67 expression level and neoadjuvant treatment response and prognosis in breast cancer based on the Neo-Bioscore staging system. Discov. Oncol..

[27] Weir C.B., Jan A. (2025). BMI Classification Percentile and Cut Off Points. StatPearls.

[28] Miller S.A., Dykes D.D., Polesky H.F. (1988). A simple salting out procedure for extracting DNA from human nucleated cells. Nucleic Acids Res..

[29] Akkiprik M., Sonmez O., Gulluoglu B.M., Caglar H.B., Kaya H., Demirkalem P., Abacioglu U., Sengoz M., Sav A., Ozer A. (2009). Analysis of p53 gene polymorphisms and protein over-expression in patients with breast cancer. Pathol. Oncol. Res..

[30] Freudenheim J.L. (2020). Alcohol’s Effects on Breast Cancer in Women. Alcohol. Res. Curr. Rev..

[31] Fan D., Xia Q., Lin D., Ma Y., Rao J., Liu L., Tang H., Xu T., Li P., Chen G. (2023). Role of breastfeeding on maternal and childhood cancers: An umbrella review of meta-analyses. J. Glob. Health.

[32] Naaman S.C., Shen S., Zeytinoglu M., Iyengar N.M. (2022). Obesity and Breast Cancer Risk: The Oncogenic Implications of Metabolic Dysregulation. J. Clin. Endocrinol. Metab..

[33] Morten B.C., Wong-Brown M.W., Scott R.J., Avery-Kiejda K.A. (2016). The presence of the intron 3 16 bp duplication poly-morphism of p53 (rs17878362) in breast cancer is associated with a low Δ40p53:p53 ratio and better outcome. Carcinogenesis.

[34] Perriaud L., Marcel V., Sagne C., Favaudon V., Guédin A., De Rache A., Guetta C., Hamon F., Teulade-Fichou M.-P., Hainaut P. (2014). Impact of G-quadruplex structures and intronic polymorphisms rs17878362 and rs1642785 on basal and ionizing radiation-induced expression of alternative p53 transcripts. Carcinogenesis.

[35] Sun Y., Hu X. (2024). Aberrant alternative splicing in cancer: Splicing events and their regulatory mechanisms (Review). Int. J. Oncol..

